# Predicting Cesarean Section Delivery in the United States Using Machine Learning: Population-Based Retrospective Study

**DOI:** 10.2196/94172

**Published:** 2026-08-21

**Authors:** Yanchao Yang

**Affiliations:** 1 School of Business and Leadership DePauw University Greencastle, IN United States

**Keywords:** c-section, cesarean delivery, machine learning, risk prediction, geographic variation, clinical decision support, obstetric care

## Abstract

**Background:**

Cesarean section (C-section) is the most common surgical procedure in the United States, yet its use varies widely across regions and institutions. Although clinical risk factors are central to delivery decisions, geographic context, health system capacity, and local practice patterns may also influence C-section use. Understanding both the determinants and predictability of C-section delivery is important for improving obstetric quality and equity.

**Objective:**

This study aims to document geographic variation in C-section use across the United States, identify maternal and county-level factors associated with C-section delivery, and evaluate the predictive performance of machine learning models across clinically defined risk groups.

**Methods:**

This population-based study used 38,133,279 US births from the 2013-2022 National Vital Statistics System Natality Detailed Files. County identifiers were linked to national county-level measures of insurance coverage, health care capacity, and socioeconomic conditions. Logistic regression models with county fixed effects were used for feature interpretation, and supervised machine learning models were used for prediction. Analyses were conducted separately for the full sample, a low-risk sample (n=17,760,772), and a high-risk sample (n=20,372,438). Predictive performance was evaluated using accuracy, precision, recall, *F*_1_-score, and area under the receiver operating characteristic curve (AUC), with 200-bootstrap 95% CIs. Temporal validation was also conducted using training on earlier years and testing on later years.

**Results:**

County-level C-section rates declined modestly from 32.35% in 2013 to 31.49% in 2022, but substantial geographic variation persisted, with consistently higher rates in the US South. In the full sample, model discrimination was good, with AUC values ranging from 0.8310 for logistic regression to 0.8401 for extreme gradient boosting (XGBoost). Predictive performance was substantially weaker in the low-risk sample (AUC 0.7246-0.7410) than in the high-risk sample (AUC 0.8404-0.8568). In the high-risk sample, XGBoost achieved the highest AUC (0.8568) and *F*_1_-score (0.7579), while random forest achieved the highest recall (0.7142). Temporal validation yielded similar results in the full sample (AUC 0.8335-0.8387), temporal low-risk sample (AUC 0.7364-0.7432), and temporal high-risk sample (AUC 0.8360-0.8479), indicating stable performance over time.

**Conclusions:**

C-section use in the United States is shaped by both maternal clinical risk and geographic context. Machine learning models perform well overall and especially well in high-risk pregnancies, but prediction is substantially more difficult in low-risk pregnancies, where discretionary and contextual influences may play a larger role. These findings support the use of risk-adjusted, context-aware prediction tools for audit, benchmarking, and clinical decision support while underscoring the need for cautious implementation, subgroup monitoring, and further external validation.

## Introduction

Cesarean section (C-section) accounts for approximately one-third of births in the United States and is the most commonly performed surgical procedure in the country [1,2]. Although C-section can be life-saving when medically indicated, both overuse and underuse raise important clinical and policy concerns. Excessive use has been associated with maternal morbidity, complications in future pregnancies, and higher health care costs, whereas insufficient use may delay necessary intervention in high-risk situations [3-5]. For this reason, improving the appropriateness of C-section use remains a central challenge in obstetric care.

The determinants of C-section delivery extend well beyond immediate clinical need. Established medical factors such as prior cesarean delivery, breech presentation, hypertensive disorders, diabetes, obesity, and multiple gestation are important [6,7], but nonmedical influences also matter. Prior research has shown that maternal socioeconomic characteristics [6,8,9], physician practice styles [10], hospital capacity [1], institutional norms [11], financial incentives [12], and broader geographic context contribute to variation in C-section use across hospitals, counties, and states, even among clinically similar patients. Persistent regional differences therefore raise concern that delivery decisions may reflect not only maternal risk but also place-based differences in care environments.

Recent advances in AI and machine learning create new opportunities to study this problem. Predictive models can help quantify predelivery C-section risk, identify the features most strongly associated with delivery mode, and evaluate whether use patterns align with underlying clinical need [13-15]. At the same time, the application of AI in obstetric care raises important concerns related to interpretability, subgroup performance, fairness, and implementation within real clinical workflows [16,17]. These issues are particularly relevant in C-section decision-making, where the balance between medical necessity, provider discretion, and institutional context is often complex.

This study addresses these issues by combining interpretable regression analysis with supervised machine learning in a large national dataset covering nearly the universe of US births from 2013 to 2022. By linking restricted-use natality records to county-level measures of health system capacity and socioeconomic context, the analysis evaluates C-section use as both a clinical and geographic phenomenon. The specific aims are to document county-level variation in C-section use, estimate the maternal and contextual correlates of C-section delivery, and compare predictive performance across the full sample and clinically defined low-risk and high-risk subgroups. In addition, temporal validation is used to assess whether model performance remains stable over time.

## Methods

### Data Source

The 2013-2022 US Natality Detailed Files from the National Vital Statistics System [18] are used as the primary data source. These files capture nearly the universe of births in the United States and are derived from official birth certificates. The data contain detailed information on maternal demographic and socioeconomic characteristics, maternal health conditions and behaviors, infant outcomes, and medical procedures associated with delivery. The large sample size and comprehensive clinical detail of the Natality data make them well suited for population-based predictive modeling. The analysis also uses the restricted-access version of the Natality data, which includes county identifiers for the mother’s county of residence. County-level information is critical for capturing the geographic context in which delivery decisions are made, as obstetric practices are shaped not only by individual clinical risk but also by local health care capacity, resource availability, and socioeconomic conditions [1]. Using county identifiers for mothers’ county of residence, the Natality data are linked to several nationally representative county-level datasets. County-level percentages of uninsured residents are obtained from the Small Area Health Insurance Estimates (SAHIE) program [19]. Measures of health care system capacity, including the supply of hospitals, physicians, and obstetrician-gynecologists, are drawn from the area health resources file (AHRF). AHRF [20] also provides county-level socioeconomic indicators, such as educational attainment and deep poverty rates. Together, these county-level measures make it possible to account for variation in local socioeconomic conditions and health care infrastructure that may influence C-section use beyond individual-level risk factors.

### Outcome Variable and Predictors

C-section is the primary outcome variable. Following the definition in the US Natality Data User’s Guide, the C-section variable in this study is coded as a binary indicator equal to 1 if the delivery was by primary or repeat cesarean section and 0 otherwise (vaginal delivery) [18]. Features observable prior to delivery are selected so that the resulting model can be applied for clinical decision support or maternal self-assessment before birth [17]. To ensure data quality and model stability, only variables with fewer than 5% missing observations are retained. For the remaining variables, missing values are handled using data imputation. Continuous variables are imputed using the sample mean, and categorical variables are imputed using the mode of the variable. Applying these selection and imputation rules yields a final feature set of 42 variables. In addition, the county identifier for the mother’s county of residence is included. This county identifier is used in the logistic regression models as a county fixed effect to account for unobserved, time-invariant county-level factors that may influence C-section use, such as local practice norms or institutional characteristics.

Binary indicator variables, coded as 1 if yes and 0 otherwise, include maternal college education, race and ethnicity indicators (Non-Hispanic White, Non-Hispanic Black, Asian, Hispanic, American Indian and Alaska Native), marital status, insurance type (Medicaid, private insurance, and self-pay), pre-pregnancy diabetes, gestational diabetes, pre-pregnancy hypertension, gestational hypertension, hypertension with eclampsia, pre-pregnancy obesity, hepatitis C, hepatitis B, syphilis, smoking during pregnancy, and use of infertility treatment. Continuous variables include maternal age, month prenatal care began, number of prenatal visits, maternal weight gain (in pounds), hospital beds per 1000 births, surgeons per 1000 births, operating rooms per 1000 births, obstetrician-gynecologists per 1000 births, physicians per 1000 births, hospitals per 1000 births, hospital bassinets per 1000 births, county-level percentage uninsured, per capita income (in 2020 US dollars), percentage of the population with 4 or more years of college education, percentage with a high school diploma, and the deep poverty rate.

### Sample Construction

Using the Natality data, 3 analytic samples are constructed: a full sample, a low-risk sample, and a high-risk sample. This stratification addresses heterogeneity in clinical risk profiles and makes it possible to assess whether predictive performance and the determinants of C-section use vary systematically across populations with substantially different baseline risks. Risk stratification is standard in obstetric research because it helps distinguish clinically appropriate C-section use from potential overuse.

The full sample includes all births with complete information on the selected covariates and consists of 38,133,279 observations. The low-risk sample includes mothers who meet all of the following criteria: maternal age 18-35 years, singleton pregnancy, no prior cesarean delivery, no pre-pregnancy diabetes, no chronic hypertension, no gestational hypertension, no eclampsia, and a pre-pregnancy BMI below 30. This group represents pregnancies for which C-section is generally less likely to be clinically necessary and includes 17,760,772 observations. The high-risk sample includes mothers who are younger than 18 or older than 35 years and who also meet at least one of the following conditions: obesity (BMI ≥30), pre-pregnancy diabetes, gestational diabetes, chronic hypertension, gestational hypertension, eclampsia, prior cesarean delivery, or multiple gestation. This group captures pregnancies with elevated medical risk and a higher expected clinical need for C-section and includes 20,372,438 observations. A small number of observations (n=69) were excluded from subgroup classification because of missing information required to determine risk status. These observations were retained in the full sample analyses but not included in the low-risk or high-risk subgroup analyses.

The logistic regression models are estimated using the full analytic sample to preserve population-level precision and maximize statistical power for coefficient estimation. By contrast, the supervised machine learning models are trained and tuned using a 10% random sample drawn within each birth year. This stratified sampling approach preserves the temporal distribution of births across the study period while substantially reducing the computational burden associated with model training, cross-validation, and hyperparameter tuning. This step is especially important for nonlinear and ensemble-based models, whose tuning procedures are computationally intensive in a dataset containing tens of millions of observations. Sampling within each year ensures that all calendar years remain proportionally represented in the machine learning training data and reduces the risk that model tuning is disproportionately influenced by years with larger numbers of observations or by shifts in the underlying population over time.

After model training and threshold selection, predictive performance is evaluated using held-out testing data. This design allows efficient model tuning while maintaining a temporally representative training structure. Accordingly, the reported performance metrics reflect out-of-sample predictive accuracy rather than in-sample fit.

### Statistical Analysis

#### Descriptive Analysis

Descriptive analyses are first conducted to characterize county-level geographic variation in C-section rates and to summarize the outcome variable and model features. County-level C-section rates in 2013 and 2022 are mapped across the United States using Tableau Public. Descriptive summary statistics for all study variables are then presented separately for the full sample, low-risk sample, and high-risk sample using Stata 17. Continuous variables and binary indicators are summarized using means and standard deviations. For binary variables, the mean corresponds to the proportion of observations with the characteristics. This approach is adopted to provide a consistent representation of feature distributions across all variables used in the predictive models and to facilitate direct comparisons of model inputs across the full, low-risk, and high-risk samples. These tables compare maternal demographic and socioeconomic characteristics, maternal health conditions, prenatal care behaviors, and county-level health system and socioeconomic characteristics across risk groups.

#### Logistic Regression

For feature interpretation, logistic regression models are estimated to examine the association between individual features and the probability of C-section delivery. Separate models are estimated for the full sample, low-risk sample, and high-risk sample. All models adjust for individual-level and county-level characteristics. County fixed effects are included to account for time-invariant differences across maternal residential areas, including persistent differences in population characteristics, health care access, and local socioeconomic conditions that may influence C-section use. Because geographic identifiers represent maternal residence rather than delivery location, these fixed effects should be interpreted as capturing broader community-level context rather than hospital-specific practice environments. Regression coefficients are reported as log-odds and interpreted using odds ratios with 95% CIs. Given the extremely large sample size, *P* values alone may identify statistically significant but practically small associations. Therefore, interpretation of logistic regression results focused primarily on the magnitude and direction of effect estimates and their potential clinical relevance rather than statistical significance alone. All logistic regression analyses were conducted using Stata 17 (StataCorp LLC).

### Machine Learning Models and Model Evaluations

For prediction and risk modeling of C-section delivery, a set of supervised machine learning models was trained separately for the full sample, the low-risk sample, and the high-risk sample. The machine learning models included standard logistic regression, ridge logistic regression with L2 regularization, lasso logistic regression with L1 regularization, random forest, and extreme gradient boosting (XGBoost). Together, these approaches span linear, regularized, and nonlinear ensemble-based methods, allowing for a comparison of predictive performance across models with differing levels of complexity and flexibility.

The analytic dataset was divided into training and testing sets using an 80/20 split, with stratification on the outcome to preserve the prevalence of C-section deliveries in both samples. Model hyperparameters were tuned using cross-validation and grid search procedures tailored to each algorithm to reduce overfitting and improve out-of-sample performance. In addition to the random train-test split, temporal validation was conducted to assess model generalizability over time. Specifically, models were trained using earlier years of data and tested on later years, providing a more realistic evaluation of how the prediction models would perform when applied prospectively to future birth cohorts.

In the clinical context, recall is an important performance metric because it measures the proportion of actual C-sections that are correctly identified by the model [7]. A low recall indicates a higher false-negative rate, meaning that cases ultimately resulting in C-section would not be flagged as high risk by the prediction model [21]. In obstetric decision-making, false negatives may be particularly consequential because delayed recognition of cases requiring surgical delivery could limit opportunities for timely clinical preparation and intervention. Therefore, model evaluation emphasized not only overall discrimination but also the model’s ability to identify true C-section cases. Classification thresholds are critical for translating predicted probabilities into binary risk classifications. For each model, predicted probabilities were generated, and candidate thresholds ranging from 0 to 1 in increments of 0.01 were evaluated. The final classification threshold was selected using the Youden J criterion, a conventional receiver operating characteristic–based method for identifying an optimal cutoff. Youden J is defined as sensitivity plus specificity minus one, and the selected threshold is the value that maximizes the combined performance of sensitivity and specificity [22]. This approach provides a data-driven cutoff that balances true-positive identification against false-positive classification while avoiding reliance on an arbitrary probability threshold such as 0.50. For the machine learning models, geographic information was incorporated at the regional level rather than the county level because of computational constraints. Model performance was further evaluated using accuracy, precision, recall, *F*_1_-score, and the area under the receiver operating characteristic curve (AUC).

### Shapley Additive Explanations Analysis

To improve model interpretability of machine learning models, Shapley additive explanations (SHAP) values from the XGBoost model were used to examine the relative contribution of individual predictors to C-section risk prediction [23,24]. The SHAP analysis provides insight into both the direction and magnitude of each predictor’s influence on predicted risk, thereby helping to identify the clinical, demographic, geographic, and institutional factors most strongly associated with model-generated C-section risk.

**Figure 1:**
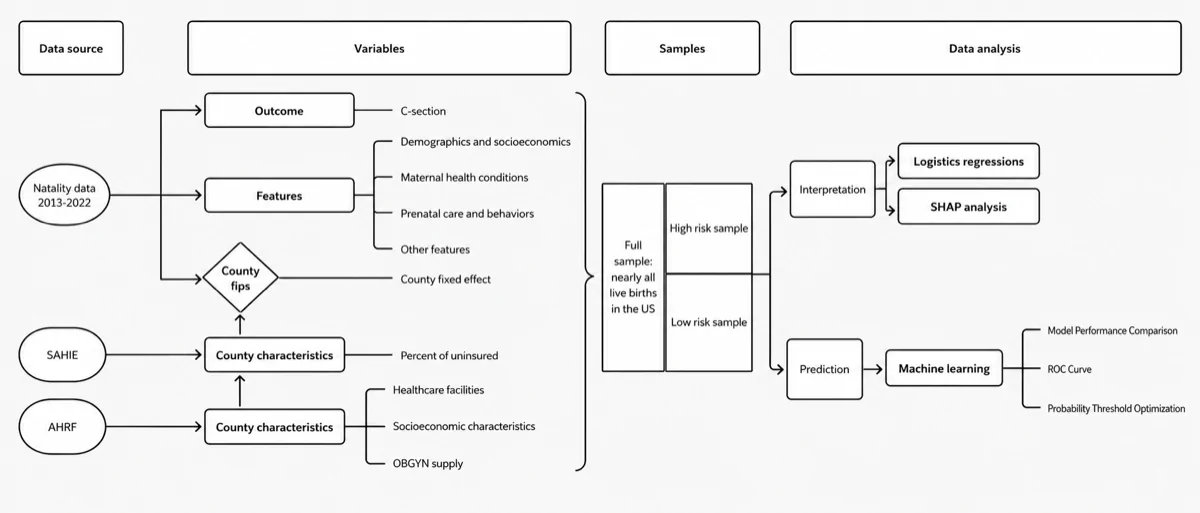
Study design and analytical framework. AHRF: area health resources file; ROC: receiver operating characteristic; SAHIE: Small Area Health Insurance Estimates; SHAP: Shapley additive explanations.

Figure 1 summarizes the study design and analytical framework of this study.

### Ethical Considerations

This study used restricted-use confidential natality data from the National Vital Statistics System (NVSS) obtained through an approved National Center for Health Statistics (NCHS) data use agreement. The study involved secondary analysis of existing data and did not include recruitment of participants, direct interaction with human participants, or collection of new information. Because this study used previously collected data and involved secondary analysis only, informed consent was not applicable. The project was reviewed by the Institutional Review Board (IRB) at DePauw University, which determined that it did not constitute human subjects research as defined under 45 CFR 46.102(e) by the US Department of Health and Human Services. Therefore, IRB approval was not required for this study. All analyses were conducted in accordance with NCHS confidentiality requirements, including restrictions on identification of individuals, data security protections, and reporting only aggregate results to protect privacy and confidentiality.

This study was reported in accordance with the TRIPOD+AI (Transparent Reporting of a multivariable prediction model for Individual Prognosis Or Diagnosis–Artificial Intelligence) statement, which provides updated reporting recommendations for clinical prediction models developed using regression or machine learning approaches [17]. The completed TRIPOD+AI checklist is provided in the Multimedia Appendix 1.

## Results

### Descriptive Analysis Results

The full summary statistics for all variables are reported in Table 1. Relative to the low-risk sample, the high-risk sample has a substantially higher C-section rate, older maternal age, and greater overall medical complexity, while the low-risk sample reflects pregnancies with systematically lower observable clinical risk by construction. These contrasts support the use of stratified analyses and provide an important descriptive foundation for interpreting both the regression and machine learning results.

**Figure 2:**
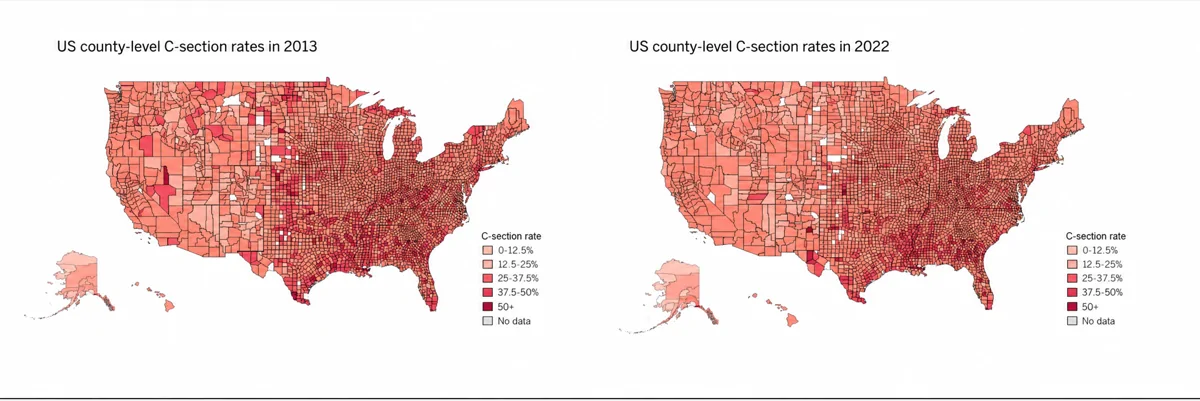
County-level C-section rates in the United States in 2013 and 2022. To protect the confidentiality and privacy of data subjects, rates are not reported for cells with fewer than 10 observations.

Figure 2 documents substantial and persistent county-level variation in C-section rates between 2013 and 2022, despite only a modest national decline from 32.35% to 31.49%. Counties in the southern United States consistently exhibit higher rates, and geographic dispersion increases over time, indicating growing heterogeneity in delivery practices across places. Table 1 shows an overall C-section rate of 32% in the full sample, compared with 16.7% in the low-risk sample and 45.4% in the high-risk sample. The high-risk sample is older on average than the low-risk sample (30.3 vs 27.2 years) and exhibits substantially greater baseline clinical complexity, whereas the low-risk sample has a lower average risk profile by construction. These descriptive differences support the use of stratified analyses and suggest that both clinical need and geographic context are important for understanding variation in C-section use.

**Table 1:** Summary statistics for the full, low-risk, and high-risk birth samples, 2013-2022.

Outcome variables		Full sample (N=38,133,279), mean (SD)	Low-risk^a^ (n=17,760,772), mean (SD)	High-risk^a^ (n=20,372,438), mean (SD)
C-section rate		0.320 (0.467)	0.167 (0.373)	0.454 (0.498)
Maternal demographic and socioeconomic characteristics				
	Mother’s age (years)	28.872 (5.850)	27.241 (4.674)	30.293 (6.377)
	College educated	0.419 (0.490)	0.437 (0.493)	0.404 (0.487)
	Non-Hispanic White	0.475 (0.499)	0.492 (0.500)	0.460 (0.498)
	Non-Hispanic Black	0.129 (0.336)	0.108 (0.310)	0.148 (0.355)
	Asian	0.059 (0.235)	0.063 (0.243)	0.054 (0.227)
	Hispanic	0.228 (0.419)	0.215 (0.411)	0.239 (0.426)
	American Indian and Alaska Native	0.007 (0.085)	0.006 (0.079)	0.008 (0.091)
	Married	0.557 (0.497)	0.559 (0.497)	0.555 (0.497)
	Medicaid	0.414 (0.493)	0.396 (0.489)	0.430 (0.495)
	Private insurance	0.483 (0.500)	0.483 (0.500)	0.484 (0.500)
	Self-pay	0.041 (0.199)	0.047 (0.211)	0.037 (0.188)
Maternal health conditions				
	Pre-pregnancy diabetes	0.009 (0.095)	—^b^	0.017 (0.130)
	Gestational diabetes	0.065 (0.247)	—	0.122 (0.327)
	Pre-pregnancy hypertension	0.020 (0.141)	—	0.038 (0.192)
	Gestational hypertension	0.069 (0.253)	—	0.129 (0.335)
	Hypertension eclampsia	0.003 (0.051)	—	0.005 (0.069)
	Pre-pregnancy obesity	0.292 (0.455)	—	0.546 (0.498)
	Hepatitis C	0.004 (0.065)	0.004 (0.065)	0.004 (0.065)
	Hepatitis B	0.002 (0.045)	0.002 (0.044)	0.002 (0.047)
	Syphilis	0.001 (0.036)	0.001 (0.033)	0.002 (0.039)
Prenatal care and behaviors				
	Month prenatal care began	2.913 (1.490)	2.916 (1.483)	2.912 (1.496)
	Number of prenatal visits	10.729 (4.166)	10.553 (3.891)	10.886 (4.384)
	Smoked during pregnancy	0.060 (0.237)	0.060 (0.237)	0.059 (0.236)
	Infertility treatment	0.019 (0.135)	0.010 (0.097)	0.026 (0.160)
	Weight gain (lbs)	29.734 (14.881)	31.705 (13.728)	28.020 (15.614)
Other factors				
	Male infant	0.512 (0.500)	0.512 (0.500)	0.512 (0.500)
	Breech presentation	0.039 (0.193)	0.027 (0.161)	0.050 (0.217)
	Previous cesarean	0.151 (0.358)	—	0.282 (0.450)
	First live birth	0.383 (0.486)	0.476 (0.499)	0.301 (0.459)
County health system characteristics				
	Hospital beds per 1000 births	242.016 (179.173)	240.404 (172.856)	243.409 (184.485)
	Surgeons per 1000 births	10.249 (8.352)	10.204 (8.314)	10.288 (8.384)
	Operating rooms per 1000 births	8.667 (7.469)	8.627 (7.392)	8.702 (7.536)
	Number of obstetricians and gynecologists per 1000 births	10.287 (7.174)	10.268 (7.117)	10.304 (7.223)
	Doctor of medicine per 1000 births	274.628 (210.039)	273.640 (209.030)	275.486 (210.910)
	Hospitals per 1000 births	1.609 (2.124)	1.601 (2.028)	1.615 (2.205)
	Hospital bassinets per 1000 births	20.643 (57.604)	21.301 (60.160)	20.064 (55.251)
County socioeconomic characteristics				
	Percent uninsured (%)	27.961 (32.407)	28.406 (32.507)	27.577 (32.317)
	Per capita income (2020 US $)	54,604 (17,564)	54,511(17,517)	54,686 (17,603)
	Population with 4+ years of college (%)	31.044 (11.001)	31.089 (10.942)	31.006 (11.051)
	Population with high school diploma (%)	87.091 (5.794)	87.118 (5.759)	87.066 (5.825)
	Deep poverty rate (%)	6.306 (2.466)	6.281 (2.437)	6.328 (2.490)

^a^Low-risk and high-risk samples are defined based on clinical risk factors.

^b^N/A: not available.

### Logistic Regression Results

Table 2 presents the logistic regression estimates for the full sample and the stratified low-risk and high-risk samples. Across all models, maternal age is positively associated with C-section delivery. College education is associated with lower odds of C-section, whereas Non-Hispanic Black mothers face higher odds across all 3 samples after adjustment for extensive maternal, clinical, and county-level characteristics. The largest associations are observed for established obstetric risk factors. In the full and high-risk samples, prior cesarean delivery and breech presentation have by far the largest coefficients, indicating their dominant role in delivery mode decisions. Additional clinical risk factors, including diabetes, hypertensive disorders, obesity, smoking during pregnancy, and infertility treatment, are also associated with higher odds of C-section, while earlier initiation of prenatal care is associated with lower odds. County-level socioeconomic variables are statistically significant but smaller in magnitude, suggesting that both clinical factors and place-based conditions contribute to variation in C-section use net of county fixed effects.

**Table 2:** Multivariable logistic regression estimates for C-section for full, low-risk, and high-risk samples with county fixed effects (2013-2022).

		Full sample^a^		Low-risk sample^a^		High-risk sample^b^	
		Log odds (SE)	*P* value	Log odds (SE)	*P* value	Log odds (SE)	*P* value
Maternal demographic and socioeconomic characteristics							
	Mother’s age	0.0528 (0.0001)	<.001	0.0626 (0.0008)	<.001	0.0405 (0.0003)	<.001
	College degree	–0.0218 (0.0011)	<.001	–0.0372 (0.0122)	<.001	–0.0126 (0.0080)	.12
	Non-Hispanic White	–0.1976 (0.0017)	<.001	–0.2594 (0.0191)	<.001	–0.1447 (0.0140)	<.001
	Non-Hispanic Black	0.0566 (0.0020)	<.001	0.0730 (0.0231)	<.001	0.0672 (0.0195)	<.001
	Asian	–0.0406 (0.0024)	<.001	–0.0303 (0.0199)	.13	–0.0598 (0.0200)	<.001
	Hispanics	–0.1048 (0.0019)	<.001	–0.0707 (0.0212)	<.001	–0.1143 (0.0169)	<.001
	American Indian and Alaska Native	–0.1461 (0.0061)	<.001	–0.1497 (0.0381)	<.001	–0.1203 (0.0261)	<.001
	Married	–0.1246 (0.0011)	<.001	–0.1515 (0.0126)	<.001	–0.0888 (0.0096)	<.001
	Medicaid	–0.0316 (0.0011)	<.001	–0.0247 (0.0141)	.08	–0.0381 (0.0129)	<.001
	Self-pay	–0.4707 (0.0026)	<.001	–0.4333 (0.0363)	<.001	–0.4903 (0.0390)	<.001
Maternal health conditions (0.0141)							
	Pre-pregnancy diabetes	0.7900 (0.0044)	<.001	—^b^	—	0.7126 (0.0106)	<.001
	Gestational diabetes	0.2717 (0.0017)	<.001	—	—	0.1695 (0.0054)	<.001
	Pre-pregnancy hypertension	0.4517 (0.0030)	<.001	—	—	0.3761 (0.0063)	<.001
	Gestational hypertension	0.4858 (0.0016)	<.001	—	—	0.3303 (0.0071)	<.001
	Hypertension eclampsia	0.8925 (0.0076)	<.001	—	—	0.7691 (0.0214)	<.001
	Pre-pregnancy obesity	0.5725 (0.0010)	<.001	—	—	0.3385 (0.0060)	<.001
	Hepatitis C	0.1289 (0.0070)	<.001	0.1959 (0.0124)	<.001	0.0893 (0.0133)	<.001
	Hepatitis B	–0.0517 (0.0099)	<.001	0.0009 (0.0191)	.96	–0.0838 (0.0191)	<.001
	Syphilis	0.0835 (0.0121)	<.001	0.1353 (0.0232)	<.001	0.0655 (0.0167)	<.001
Prenatal care and behaviors							
	Month prenatal care began	–0.0077 (0.0003)	<.001	–0.0013 (0.0013)	.32	–0.0135 (0.0012)	<.001
	Number of prenatal visits	0.0080 (0.0001)	<.001	0.0058 (0.0009)	<.001	0.0094 (0.0007)	<.001
	Smoke during pregnancy	0.1297 (0.0020)	<.001	0.1234 (0.0056)	<.001	0.1469 (0.0049)	<.001
	Infertility treatment	0.7160 (0.0030)	<.001	0.3529 (0.0085)	<.001	0.7853 (0.0117)	<.001
	Weight gain	0.0113 (0.0000)	<.001	0.0112 (0.0001)	<.001	0.0112 (0.0001)	<.001
Other factors							
	Male infant	0.1598 (0.0009)	<.001	0.2039 (0.0027)	<.001	0.1320 (0.0016)	<.001
	Breech	4.0914 (0.0035)	<.001	4.5379 (0.0363)	<.001	3.7199 (0.0227)	<.001
	Previous cesarean delivery	3.8167 (0.0015)	<.001	—	—	3.6040 (0.0315)	<.001
	First live birth	1.1046 (0.0010)	<.001	1.1792 (0.0249)	<.001	1.0646 (0.0138)	<.001
County health system characteristics							
	Hospital beds per 1000 births	–0.0001 (0.0000)	<.001	–0.0001 (0.0001)	.32	–0.0000 (0.0000)	>.99
	Surgeons per 1000 births	–0.0010 (0.0003)	<.001	–0.0010 (0.0024)	.68	–0.0007 (0.0015)	.64
	Operating rooms per 1000 births	–0.0003 (0.0002)	.13	–0.0004 (0.0009)	.66	–0.0004 (0.0005)	.42
	OB-GYNs^c^ per 1000 births	0.0022 (0.0003)	<.001	0.0037 (0.0025)	.14	0.0013 (0.0015)	.39
	MDs^d^ per 1000 births	0.0000 (0.0000)	<.001	0.0001 (0.0001)	.32	–0.0000 (0.0001)	>.99
	Hospitals per 1000 births	–0.0030 (0.0012)	.01	–0.0022 (0.0055)	.69	–0.0036 (0.0030)	.23
	Hospital bassinets per 1000 births	0.0001 (0.0000)	<.001	0.0000 (0.0001)	>.99	0.0001 (0.0000)	<.001
County socioeconomic characteristics							
	Percent uninsured (%)	–0.0005 (0.0000)	<.001	–0.0007 (0.0001)	<.001	–0.0003 (0.0001)	.003
	Per capita income (2020 $)	–0.0000 (0.0000)	<.001	–0.0000 (0.0000)	<.001	–0.0000 (0.0000)	<.001
	Population with 4+ years of college	–0.0168 (0.0004)	<.001	–0.0128 (0.0061)	.04	–0.0182 (0.0045)	<.001
	Population with high school diploma	–0.0319 (0.0005)	<.001	–0.0408 (0.0051)	<.001	–0.0268 (0.0036)	<.001
	Deep poverty (%)	0.0062 (0.0005)	<.001	0.0085 (0.0035)	.02	0.0045 (0.0018)	.01
	Observations, n	38,133,279	—	17,760,772	—	20,372,438	—

^a^All models include county fixed effects.

^b^Not available.

^c^OB-GYN: obstetricians and gynecologists.

^d^MD: doctor of medicine.

### Predictive Performance of Machine Learning Models

**Figure 3:**
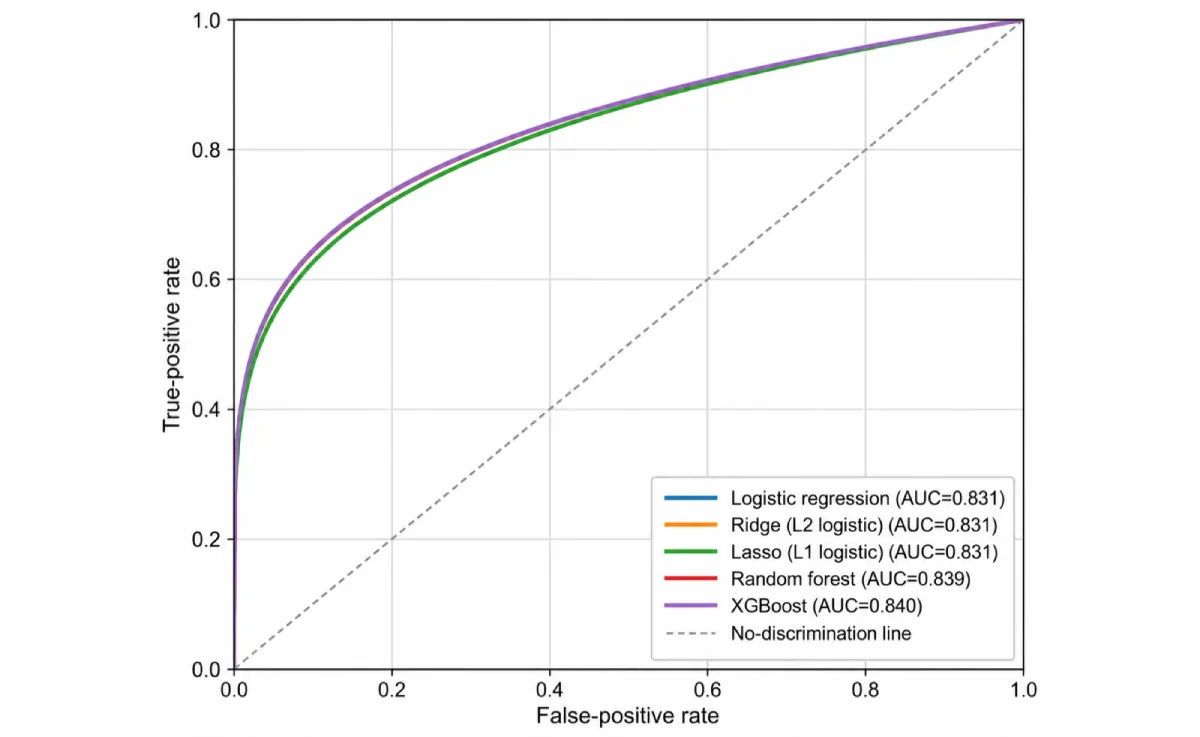
Receiver operating characteristic curves comparing prediction models in the full sample. AUC: area under the receiver operating characteristic curve; XGBoost: extreme gradient boosting.

Table 3 and Figure 3 summarize predictive performance for the full sample. In the full sample, all 5 models perform well, with AUC values ranging from 0.8310 for logistic regression and ridge logistic regression to 0.8401 for XGBoost. The 3 logistic specifications yield nearly identical results, whereas ensemble methods provide modest gains in discrimination. XGBoost achieves the highest AUC (0.8401), recall (0.6828), and *F*_1_-score (0.6817), while logistic regression and ridge logistic regression yield the highest accuracy (0.7982). Random forest performs similarly, with an AUC of 0.8394 and an *F*_1_-score of 0.6810.

Predictive performance is substantially weaker in the low-risk sample.

**Figure 4:**
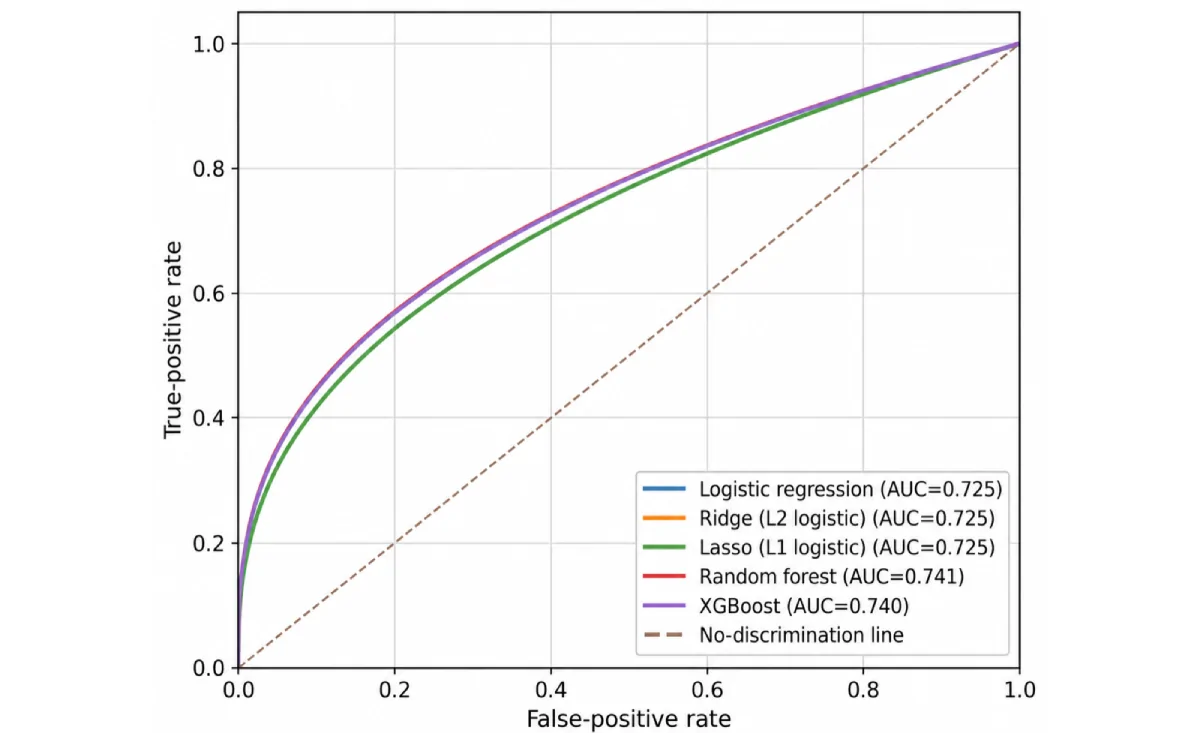
Receiver operating characteristic curves comparing prediction models in the low-risk sample. AUC: area under the receiver operating characteristic curve; XGBoost: extreme gradient boosting.

Table 4 and Figure 4 summarize that predictive performance is notably weaker in the low-risk sample. AUC values range from 0.7246 to 0.7410, substantially below those observed in the full sample and the high-risk sample. Precision remains low across all models, ranging from 0.2888 to 0.2990, which highlights the difficulty of identifying C-sections in pregnancies with relatively limited baseline clinical risk. Random forest achieves the highest AUC (0.7410), whereas XGBoost yields the highest accuracy (0.6867), the highest *F*_1_-score (0.4105), and a recall of 0.6543. These results suggest that prediction in low-risk pregnancies remains challenging even with more flexible machine learning approaches.

**Table 3:** Predictive performance of machine learning models for cesarean delivery (full sample).

Models	Threshold	Accuracy (95% CI)	Precision (95% CI)	Recall (95% CI)	*F*_1_-score (95% CI)	AUC^a^ (95% CI)
Logistic regression	0.310	0.7982 (0.7974-0.7991)	0.6955 (0.6939-0.6971)	0.6581 (0.6560-0.6600)	0.6763 (0.6748-0.6777)	0.8310 (0.8299-0.8320)
Ridge (L2 logistic)	0.310	0.7982 (0.7974-0.7991)	0.6955 (0.6939-0.6971)	0.6581 (0.6560-0.6600)	0.6763 (0.6748-0.6777)	0.8310 (0.8299-0.8320)
Lasso (L1 logistic)	0.309	0.7977 (0.7969-0.7986)	0.6939 (0.6924-0.6954)	0.6594 (0.6573-0.6613)	0.6762 (0.6747-0.6777)	0.8311 (0.8299-0.8320)
Random forest	0.467	0.7970 (0.7961-0.7979)	0.6855 (0.6839-0.6870)	0.6765 (0.6745-0.6783)	0.6810 (0.6796-0.6823)	0.8394 (0.8382-0.8404)
XGBoost^b^	0.301	0.7957 (0.7949-0.7965)	0.6805 (0.6789-0.6820)	0.6828 (0.6809-0.6844)	0.6817 (0.6802-0.6830)	0.8401 (0.8389-0.8411)

^a^AUC: area under the receiver operating characteristic curve.

^b^XGBoost: extreme gradient boosting.

**Table 4:** Predictive performance of machine learning models for cesarean delivery (low-risk sample).

Models	Threshold	Accuracy (95% CI)	Precision (95% CI)	Recall (95% CI)	*F*_1_-score (95% CI)	AUC^a^ (95% CI)
Logistic regression	0.170	0.6777 (0.6761-0.6795)	0.2889 (0.2871-0.2907)	0.6387 (0.6354-0.6426)	0.3978 (0.3957-0.4001)	0.7246 (0.7222-0.7271)
Ridge (L2 logistic)	0.170	0.6777 (0.6761-0.6795)	0.2889 (0.2871-0.2907)	0.6387 (0.6354-0.6426)	0.3978 (0.3957-0.4001)	0.7246 (0.7222-0.7271)
Lasso (L1 logistic)	0.170	0.6777 (0.6761-0.6795)	0.2888 (0.2871-0.2907)	0.6385 (0.6351-0.6424)	0.3978 (0.3956-0.3999)	0.7246 (0.7223-0.7271)
Random forest	0.480	0.6846 (0.6830-0.6862)	0.2981 (0.2964-0.2998)	0.6585 (0.6542-0.6629)	0.4104 (0.4081-0.4129)	0.7410 (0.7388-0.7435)
XGBoost^b^	0.171	0.6867 (0.6852-0.6881)	0.2990 (0.2974-0.3009)	0.6543 (0.6505-0.6583)	0.4105 (0.4084-0.4131)	0.7396 (0.7375-0.7419)

^a^AUC: area under the receiver operating characteristic curve.

^b^XGBoost: extreme gradient boosting.

**Figure 5:**
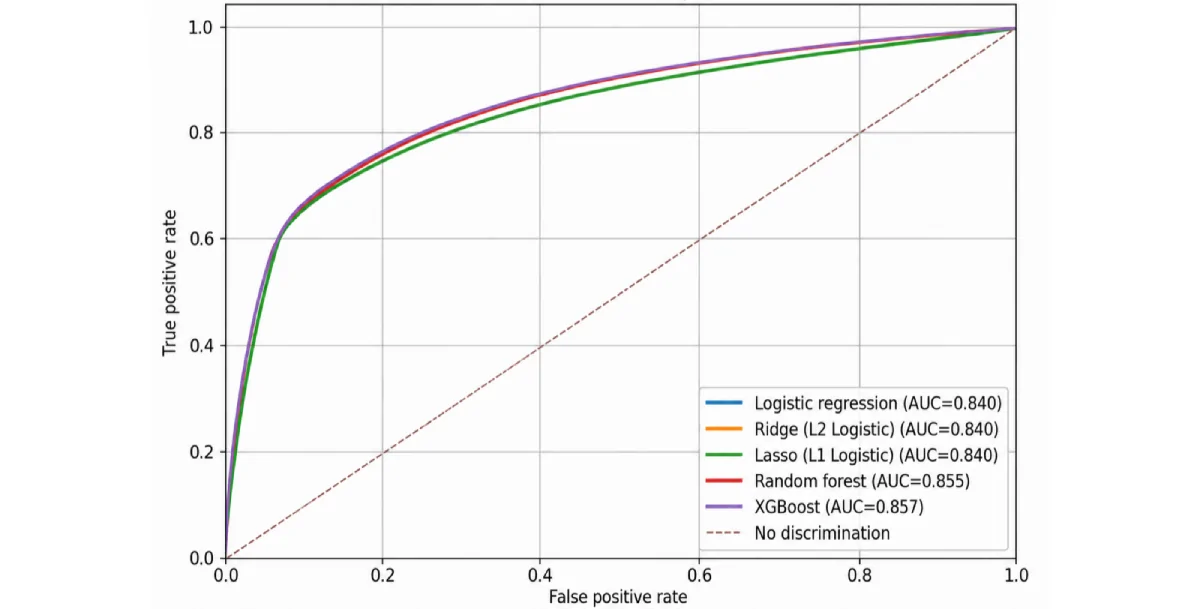
Receiver operating characteristic curves comparing prediction models in the high-risk sample. AUC: area under the receiver operating characteristic curve; ROC: Receiver operating characteristic curve; XGBoost: extreme gradient boosting.

Table 5 and Figure 5 presents by contrast, that model performance improves substantially in the high-risk sample. AUC values range from 0.8404 to 0.8568, and all models achieve substantially higher precision and *F*_1_-scores than in the low-risk sample. XGBoost delivers the strongest overall discrimination, with the highest AUC (0.8568), the highest accuracy (0.7947), and the highest *F*_1_-score (0.7579). Random forest achieves the highest recall (0.7142), while the logistic models retain slightly higher precision, peaking at 0.8220 for lasso logistic regression. Taken together, the results indicate that C-section delivery is considerably more predictable among high-risk births than among low-risk births, consistent with the greater role of identifiable clinical risk factors in high-risk pregnancies.

Temporal validation produced results that were broadly consistent with the main analysis and showed stable model performance over time (Tables S1-S3 in Multimedia Appendix 2). In the full sample, discrimination remained good, with AUC values ranging from 0.8335 for logistic regression, ridge, and lasso to 0.8387 for XGBoost, while random forest achieved an AUC of 0.8379 (Table S1 in Multimedia Appendix 2). Performance was substantially weaker in the low-risk sample, where AUC values ranged from 0.7364 for logistic regression and ridge to 0.7432 for XGBoost, with random forest at 0.7425 (Table S2 in Multimedia Appendix 2). By contrast, performance remained stronger in the high-risk sample, where AUC values ranged from 0.8360 for logistic regression, ridge, and lasso to 0.8479 for random forest, while XGBoost achieved an AUC of 0.8467 (Table S3 in Multimedia Appendix 2). Taken together, these temporal results reinforce the main finding that C-section delivery is more predictable in high-risk pregnancies than in low-risk pregnancies and suggest that the relative ranking of model performance is stable across time.

**Table 5:** Predictive performance of machine learning models for cesarean delivery (high-risk sample).

Models	Threshold	Accuracy (95% CI)	Precision (95% CI)	Recall (95% CI)	*F*_1_-score (95% CI)	AUC^a^ (95% CI)
Logistic regression	0.440	0.7897 (0.7885-0.7909)	0.8214 (0.8199-0.8227)	0.6869 (0.6850-0.6891)	0.7482 (0.7468-0.7498)	0.8404 (0.8392-0.8418)
Ridge (L2 logistic)	0.440	0.7897 (0.7885-0.7909)	0.8214 (0.8199-0.8227)	0.6869 (0.6850-0.6891)	0.7482 (0.7468-0.7498)	0.8404 (0.8392-0.8418)
Lasso (L1 logistic)	0.441	0.7897 (0.7886-0.7909)	0.8220 (0.8205-0.8233)	0.6862 (0.6842-0.6884)	0.7480 (0.7466-0.7496)	0.8404 (0.8393-0.8418)
Random forest	0.460	0.7911 (0.7900-0.7922)	0.8045 (0.8027-0.8061)	0.7142 (0.7121-0.7161)	0.7567 (0.7552-0.7582)	0.8550 (0.8539-0.8562)
XGBoost^b^	0.451	0.7947 (0.7935-0.7960)	0.8172 (0.8155-0.8187)	0.7066 (0.7046-0.7087)	0.7579 (0.7564-0.7595)	0.8568 (0.8558-0.8581)

^a^AUC: area under the receiver operating characteristic curve.

^b^XGBoost: extreme gradient boosting.

### SHAP Analysis Results

**Figure 6:**
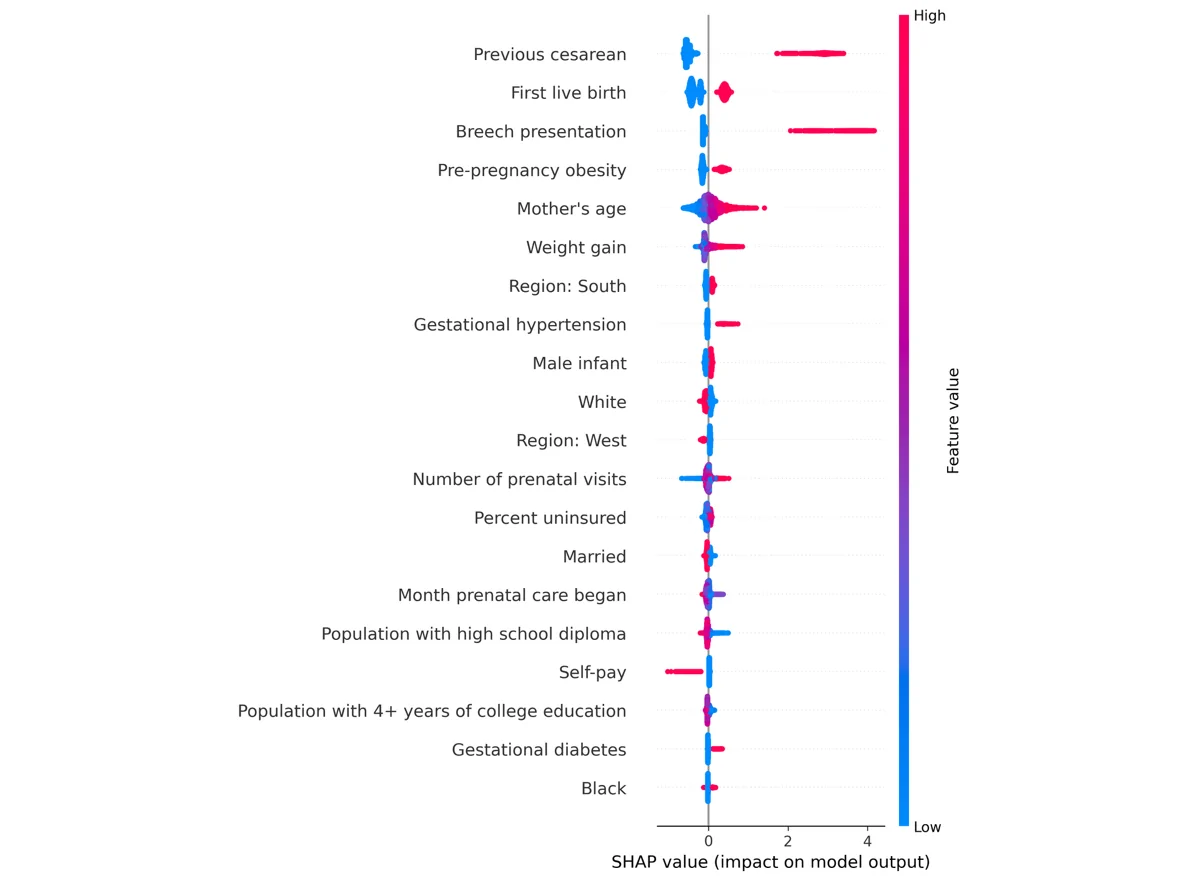
SHAP summary plot for the extreme gradient boosting model in the full sample. Features are ranked by mean absolute SHAP value; points to the right indicate higher predicted probability of C-section, and points to the left indicate lower predicted probability. SHAP: Shapley additive explanations.

Figure 6 presents SHAP summary plots for the XGBoost model in the full sample. As shown in the figure, previous cesarean delivery and breech presentation are the 2 most influential predictors, followed by first live birth, pre-pregnancy obesity, maternal age, and weight gain. Higher values of these clinical risk factors are generally associated with positive SHAP values, indicating a greater predicted probability of C-section. Several contextual and demographic variables, including region of residence, prenatal care use, insurance-related measures, and marital status, also contribute to model predictions, but their effects are smaller in magnitude than those of major obstetric risk factors.

**Figure 7:**
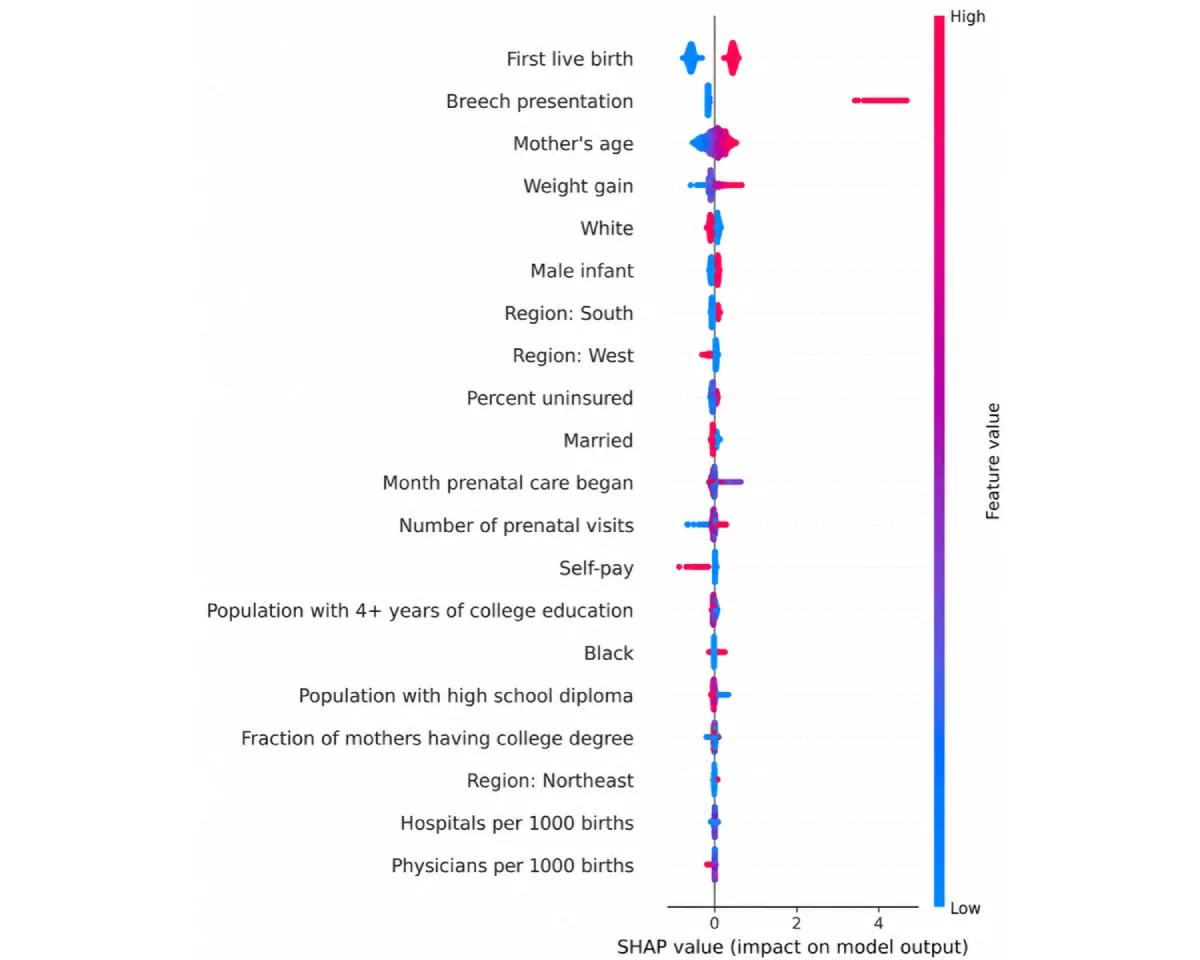
SHAP summary plot for the extreme gradient boosting model in the low-risk sample. Features are ranked by mean absolute SHAP value; points to the right indicate higher predicted probability of C-section, and points to the left indicate lower predicted probability. SHAP: Shapley additive explanations.

In the low-risk sample (Figure 7), breech presentation remains highly influential, but the overall importance ranking shifts toward factors such as first live birth, maternal age, weight gain, and selected demographic and regional variables. Compared with the full sample and high-risk sample, the low-risk SHAP profile is more diffuse, with smaller separation between the top predictors and the remaining features. This pattern is consistent with the weaker predictive performance observed in Table 4 and suggests that, in low-risk pregnancies, C-section use is shaped by a broader mix of smaller clinical, demographic, and contextual influences rather than by a small number of dominant medical risk factors.

**Figure 8:**
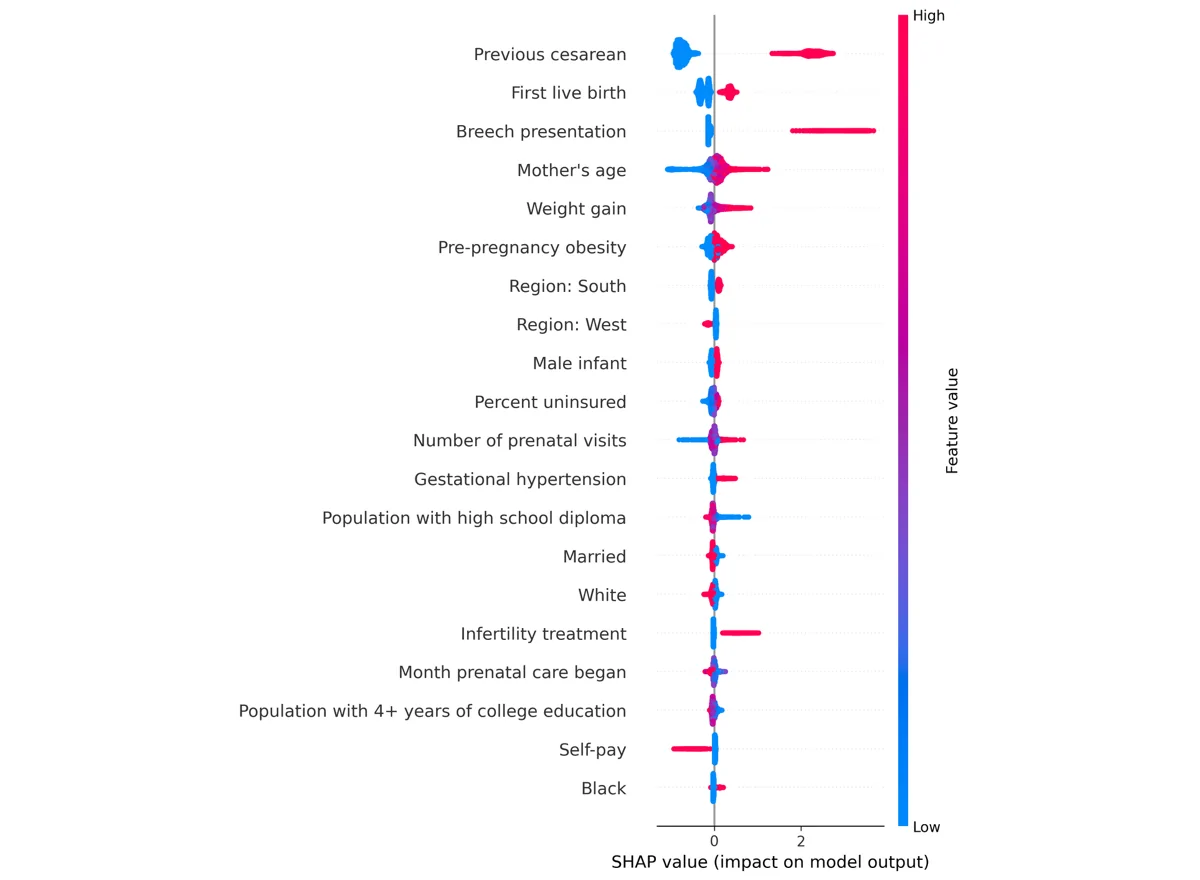
SHAP summary plot for the extreme gradient boosting model in the high-risk sample. Notes: Features are ranked by mean absolute SHAP value; points to the right indicate higher predicted probability of C-section, and points to the left indicate lower predicted probability. SHAP: Shapley additive explanations.

In the high-risk sample (Figure 8), previous cesarean delivery and breech presentation again emerge as the strongest predictors, followed by first live birth, maternal age, weight gain, pre-pregnancy obesity, and gestational hypertension. Infertility treatment also appears among the top predictors in this subgroup. The high-risk SHAP profile is more concentrated around a small number of clinically salient features, which aligns with the stronger model performance reported in Table 5.

## Discussion

### Principal Findings

This study examined geographic variation in C-section use in the United States, estimated the maternal and contextual factors associated with C-section delivery, and evaluated predictive performance across the full sample and clinically defined low-risk and high-risk subgroups. Overall, 3 main findings emerge. First, substantial county-level geographic variation in C-section rates persisted between 2013 and 2022 despite only a modest national decline in the overall rate. Second, both the regression and machine learning results indicate that C-section use is shaped by a combination of strong clinical risk factors and broader contextual influences. Third, predictive performance differed meaningfully across risk groups. Machine learning models performed well in the full sample and especially well in the high-risk sample, but performance was notably weaker in the low-risk sample. Taken together, these findings suggest that C-section delivery is more predictable when clear medical indications are present and less predictable when discretionary, institutional, and contextual influences may play a larger role.

### Comparison With Prior Work

These findings are consistent with prior research showing that large variation in C-section use persists across hospitals, counties, and states even among clinically similar births [25,26]. Earlier studies have emphasized the importance of physician practice styles, institutional norms, financial incentives, and local health system characteristics in shaping obstetric care beyond maternal medical need alone [25,27,28]. The present study extends that literature by combining individual-level natality records with county-level health system and socioeconomic measures in a national predictive framework. The regression results reinforce the central importance of established obstetric risk factors. Prior cesarean delivery, breech presentation, hypertensive disorders, diabetes, and obesity remained among the strongest correlates of C-section use, which is consistent with the broader obstetric literature [29]. At the same time, demographic and socioeconomic characteristics remained associated with C-section delivery even after adjustment for extensive clinical covariates and county fixed effects. This pattern suggests that C-section use cannot be understood solely as a function of clinical need.

The subgroup findings are especially important. The substantially weaker predictive performance in the low-risk sample should not be interpreted simply as a modeling limitation. Rather, it likely reflects the fact that C-section decisions among low-risk pregnancies are less strongly determined by observable predelivery clinical factors alone. Previous studies have documented substantial variation in low-risk cesarean delivery rates across hospitals, suggesting that institutional factors and practice patterns contribute importantly to delivery decisions beyond patient-level risk factors [25].

The temporal validation results further strengthen the interpretation of the main findings. Model performance remained broadly similar when training was conducted on earlier years and testing on later years, and the same basic pattern persisted: performance was strongest in the high-risk sample, intermediate in the full sample, and weakest in the low-risk sample. Temporal validation is an important approach for evaluating the generalizability of clinical prediction models because performance may change when models are applied to future populations or evolving clinical environments [30]. This stability over time suggests that the central findings are not an artifact of a single random split.

### Implications

Several implications follow from these results. From a clinical perspective, the stronger predictive performance observed in the high-risk sample suggests that risk prediction tools may be most useful in settings where medical complexity is already elevated. In such cases, prediction models may support care planning, triage, and communication by providing structured estimates of likely delivery mode based on information available before birth. However, the weaker performance in the low-risk sample suggests that algorithmic outputs should be interpreted much more cautiously in pregnancies where clinical need is less clearly defined.

From a health system perspective, the persistent geographic variation observed in county-level C-section rates indicates that interventions focused only on maternal-level risk factors are unlikely to be sufficient. Context-aware prediction may therefore be most useful not as a substitute for physician judgment, but as a tool for audit, benchmarking, and quality review. For example, hospitals or regions with observed C-section rates that differ substantially from risk-adjusted expectations may warrant closer review of institutional practices, staffing patterns, or local norms of care.

In practice, a context-aware prediction tool could be used in several ways. At the patient level, it could support shared decision-making by providing a structured estimate of C-section risk based on pre-delivery characteristics while still leaving room for clinical judgment and patient preferences. At the hospital or health system level, it could be used for audit and benchmarking by comparing observed C-section use with risk-adjusted expected rates across facilities, counties, or regions. It could also support quality review by helping identify settings in which C-section use appears systematically higher or lower than would be expected given the underlying case mix.

AI-based prediction in obstetric care should also be evaluated from a human-centered implementation perspective rather than solely based on discrimination or overall accuracy [16]. In practice, a model with acceptable predictive performance may still worsen inequities if it produces systematically different errors across racial, ethnic, or insurance groups, or if it is introduced into clinical workflows without adequate transparency, clinician oversight, and patient communication. From this perspective, fairness should be understood not only as a statistical property of the model, but also as a feature of how the tool is embedded in decision-making, how its outputs are interpreted, and whether it supports or undermines equitable care. Relatedly, governance of clinical AI should extend beyond technical performance metrics to include accountability, documentation, subgroup monitoring, workflow integration, and institutional review of downstream effects. For a context-aware C-section prediction tool, this means that future implementation should include fairness audits, calibration checks across patient subgroups, and ongoing hospital-level monitoring to ensure that the model supports appropriate care without reinforcing existing disparities.

The contrast in predictive performance between low-risk and high-risk pregnancies requires careful interpretation. The higher AUC observed in the high-risk sample is partly expected because this group was defined based on the presence of clinical characteristics that are among the strongest predictors of C-section delivery, particularly prior cesarean delivery. Conversely, the low-risk sample was constructed by excluding these major clinical risk factors, which mechanically reduces the amount of predictive information available to the model. Thus, the lower AUC in the low-risk sample should not be interpreted solely as evidence that delivery decisions are more discretionary in these pregnancies. Rather, the observed difference likely reflects both the reduced predictive signal resulting from sample construction and the possibility that nonclinical factors play a larger role when clear medical indications are less prominent. Future studies should consider alternative risk stratification approaches and incorporate additional measures of provider, hospital, and patient-level factors to further distinguish these mechanisms.

### Limitations

Several limitations should be acknowledged. First, this was an observational study and does not support causal inference regarding the effects of individual, institutional, or geographic factors on C-section use. Second, although the natality files provide unusually rich national coverage, they do not fully capture all determinants of delivery decisions. Important factors such as physician preferences, hospital-specific protocols, detailed patient preferences, and intrapartum events are only partially observed or not observed in the data.

Third, the prediction target represents observed C-section delivery rather than clinical necessity. Consequently, the models may capture both clinically appropriate decision-making and existing variation in practice patterns. Although this feature makes the models useful for understanding and benchmarking current care patterns, additional research is needed to develop prediction frameworks aligned more directly with clinical appropriateness.

Fourth, the very large sample size increases statistical power to the point that small coefficients may be statistically significant even when their practical importance is limited. Therefore, interpretation should focus on the magnitude and clinical relevance of effect estimates rather than statistical significance alone. Fifth, although temporal validation was conducted, external validation in other clinical datasets or care settings is still needed before these models can be considered for broader implementation. Finally, while this study discusses fairness and governance considerations, it does not provide a comprehensive subgroup fairness audit. Future work should examine whether discrimination, calibration, and threshold-based error rates differ systematically across racial, ethnic, socioeconomic, and geographic groups. Sixth, geographic measures were constructed using maternal county of residence rather than delivery location. Although residential geography provides important information on community context and health care access, it may not accurately represent the facility-level environment where delivery decisions occur. Future research linking birth records with hospital-level information may help further disentangle community-level and institutional contributors to C-section variation.

### Conclusions

C-section use in the United States remains shaped by both maternal clinical risk and geographic context. The results indicate that machine learning models can predict C-section delivery reasonably well overall and especially well among high-risk pregnancies, but they are less effective in low-risk settings where discretionary and contextual influences may be more prominent. These findings suggest that prediction tools may be most useful as risk-adjusted, context-aware supports for audit, benchmarking, and clinical decision support rather than as replacements for physician judgment. More broadly, the persistence of geographic variation after extensive adjustment underscores the need for policies and clinical strategies that address institutional and regional drivers of C-section use in addition to individual maternal risk.

## Appendix

Multimedia Appendix 1 TRIPOD–AI checklist.

Multimedia Appendix 2 Temporal predictive performance.

## Abbreviations

AHRF: area health resources file
AUC: area under the receiver operating characteristic curve
CDC: Centers for Disease Control and Prevention
C-section: cesarean section
IRB: Institutional Review Board
MD: doctor of medicine
NCHS: National Center for Health Statistics
NVSS: National Vital Statistics System
ROC: receiver operating characteristic
SAHIE: Small Area Health Insurance Estimates
SHAP: Shapley additive explanations
TRIPOD+AI: Transparent Reporting of a multivariable prediction model for Individual Prognosis Or Diagnosis–Artificial Intelligence
XGBoost: extreme gradient boosting

## Data Availability

The natality data used in this study are publicly available from the US Centers for Disease Control and Prevention (CDC) National Center for Health Statistics. Access to restricted-use files containing geographic identifiers requires approval through a formal data use agreement and cannot be publicly shared. County-level variables were constructed from publicly available data sources. The statistical code used to generate the results is available from the author upon reasonable request.
