# Supplementary material for: Predicting Cesarean Section Delivery in the United States Using Machine Learning: Population-Based Retrospective Study
Source: JMIR Form Res. 2026 Aug 21;10:e94172. doi: 10.2196/94172 (PMC13545532; doi:10.2196/94172)
Supplement: Supplementary file 1 [file formative_v10i1e94172_app1.docx]

**Multimedia Appendix 2. TRIPOD+AI Checklist – C-section Machine Learning Prediction Study**

| TRIPOD+AI Item | Requirement | Manuscript Location | Status |
| --- | --- | --- | --- |
| Title | The manuscript title identifies C-section prediction and machine learning methods. | Title page; Methods | Yes |
| Abstract | Structured abstract reports background, objective, methods, results, and conclusions relevant to prediction modeling. | Abstract | Yes |
| Background and objectives | Clinical rationale, geographic variation, and prediction objectives are described. | Introduction; Study aims | Yes |
| Data source | Restricted-use 2013–2022 NVSS Natality Detailed Files are described, including county linkage. | Methods: Data Source | Yes |
| Study population | Eligibility criteria and construction of full, low-risk, and high-risk samples are reported. | Methods: Sample Construction | Yes |
| Outcome definition | C-section delivery is defined as a binary outcome using natality data definitions. | Methods: Outcome Variable | Yes |
| Predictors | Maternal demographic, clinical, behavioral, and county-level predictors are described. | Methods: Outcome Variable and Predictors | Yes |
| Missing data | Missing-value handling and inclusion criteria are reported. | Methods: Outcome Variable and Predictors | Yes |
| Model development | Logistic regression, ridge, lasso, random forest, and XGBoost models are described. | Methods: Machine Learning Models and Model Evaluations | Yes |
| Model validation | Random train-test evaluation and temporal validation using earlier years for training and later years for testing are described. | Methods; Results | Yes |
| Performance measures | Accuracy, precision, recall, F1 score, and AUC with bootstrap confidence intervals are reported. | Methods; Results | Yes |
| Model interpretation | SHAP-based interpretation of XGBoost predictions is included with methodological citations. | Methods: SHAP Analysis; Results | Yes |
| Fairness and subgroup analysis | Performance differences across low-risk/high-risk groups and implications for fairness are discussed. | Results; Discussion | Yes |
| Clinical implementation considerations | Limitations of observed C-section prediction versus clinical necessity and need for cautious implementation are discussed. | Discussion; Limitations | Yes |
| Transparency and reporting | TRIPOD+AI checklist should be submitted as supplementary material with manuscript. | Appendix/Supplementary material | To upload |
