# Supplementary material for: Predicting Cesarean Section Delivery in the United States Using Machine Learning: Population-Based Retrospective Study
Source: JMIR Form Res. 2026 Aug 21;10:e94172. doi: 10.2196/94172 (PMC13545532; doi:10.2196/94172)
Supplement: Supplementary file 2 [file formative_v10i1e94172_app2.docx]

**Multimedia Appendix 1.** **Temporal Prediction Performance Tables**

**Appendix Table A1. Temporal Predictive Performance of Machine Learning Models in the Full Sample**

| **Model** | **Threshold** | **Accuracy  (95% CI)** | **Precision (95% CI)** | **Recall (95% CI)** | **F1 Score (95% CI)** | **AUC (95% CI)** |
| --- | --- | --- | --- | --- | --- | --- |
| Logistic Regression | 0.310 | 0.7934  (0.7925-0.7943) | 0.6778 (0.6762-0.6795) | 0.6772 (0.6752-0.6791) | 0.6775 (0.6760-0.6790) | 0.8335 (0.8325-0.8346) |
| Ridge  (L2 Logistic) | 0.310 | 0.7934  (0.7925-0.7943) | 0.6778 (0.6762-0.6795) | 0.6772 (0.6752-0.6791) | 0.6775 (0.6760-0.6790) | 0.8335 (0.8325-0.8346) |
| Lasso  (L1 Logistic) | 0.310 | 0.7934  (0.7926-0.7944) | 0.6779 (0.6762-0.6795) | 0.6773 (0.6752-0.6792) | 0.6776 (0.6760-0.6791) | 0.8336 (0.8326-0.8347) |
| Random Forest | 0.465 | 0.7914  (0.7905-0.7924) | 0.6691  (0.6675-0.6708) | 0.6908 (0.6890-0.6926) | 0.6798 (0.6783-0.6811) | 0.8379 (0.8368-0.8390) |
| XGBoost | 0.310 | 0.7953  (0.7943-0.7962) | 0.6797 (0.6780-0.6813) | 0.6830 (0.6811-0.6850) | 0.6814 (0.6799-0.6828) | 0.8387 (0.8377-0.8398) |

*Notes: Models were trained using 2013-2020 births, threshold selection and validation were conducted using 2021 births, and final predictive performance was evaluated using 2022 births. Values are presented as point estimates with 200-bootstrap 95% confidence intervals.*

**Appendix Table A2. Temporal Predictive Performance of Machine Learning Models in the Low-Risk Sample**

| **Model** | **Threshold** | **Accuracy  (95% CI)** | **Precision (95% CI)** | **Recall (95% CI)** | **F1 Score (95% CI)** | **AUC (95% CI)** |
| --- | --- | --- | --- | --- | --- | --- |
| Logistic Regression | 0.170 | 0.6775  (0.6760-0.6793) | 0.2840 (0.2823-0.2859) | 0.6617 (0.6573-0.6658) | 0.3974 (0.3953-0.3998) | 0.7364 (0.7339-0.7388) |
| Ridge  (L2 Logistic) | 0.170 | 0.6775  (0.6760-0.6794) | 0.2840 (0.2823-0.2859) | 0.6617 (0.6573-0.6658) | 0.3974 (0.3953-0.3998) | 0.7364 (0.7339-0.7388) |
| Lasso  (L1 Logistic) | 0.170 | 0.6773  (0.6757-0.6791) | 0.2838 (0.2822-0.2856) | 0.6619 (0.6574-0.6658) | 0.3973 (0.3952-0.3997) | 0.7365 (0.7340-0.7389) |
| Random Forest | 0.495 | 0.7500  (0.7486-0.7515) | 0.3316  (0.3293-0.3337) | 0.5465 (0.5418-0.5506) | 0.4127 (0.4100-0.4153) | 0.7425 (0.7401-0.7447) |
| XGBoost | 0.175 | 0.7019  (0.7003-0.7036) | 0.2988 (0.2969-0.3007) | 0.6344 (0.6304-0.6382) | 0.4062 (0.4039-0.4087) | 0.7432 (0.7407-0.7454) |

*Notes: Models were trained using 2013-2020 births, threshold selection and validation were conducted using 2021 births, and final predictive performance was evaluated using 2022 births. Values are presented as point estimates with 200-bootstrap 95% confidence intervals.*

**Appendix Table A3. Temporal Predictive Performance of Machine Learning Models in the High-Risk Sample**

| **Model** | **Threshold** | **Accuracy  (95% CI)** | **Precision (95% CI)** | **Recall (95% CI)** | **F1 Score (95% CI)** | **AUC (95% CI)** |
| --- | --- | --- | --- | --- | --- | --- |
| Logistic Regression | 0.440 | 0.7864  (0.7852-0.7875) | 0.8034 (0.8015-0.8050) | 0.6765 (0.6746-0.6785) | 0.7345 (0.7330-0.7359) | 0.8360 (0.8348-0.8370) |
| Ridge  (L2 Logistic) | 0.440 | 0.7864  (0.7852-0.7875) | 0.8034 (0.8015-0.8050) | 0.6765 (0.6746-0.6785) | 0.7345 (0.7330-0.7359) | 0.8360 (0.8348-0.8370) |
| Lasso  (L1 Logistic) | 0.440 | 0.7864  (0.7852-0.7875) | 0.8034 (0.8014-0.8049) | 0.6766 (0.6747-0.6786) | 0.7345 (0.7331-0.7359) | 0.8360 (0.8348-0.8370) |
| Random Forest | 0.435 | 0.7825  (0.7814-0.7836) | 0.7627  (0.7606-0.7643) | 0.7289 (0.7270-0.7306) | 0.7454 (0.7441-0.7467) | 0.8479 (0.8467-0.8489) |
| XGBoost | 0.445 | 0.7890  (0.7879-0.7901) | 0.8007 (0.7988-0.8024) | 0.6882 (0.6863-0.6900) | 0.7402 (0.7388-0.7416) | 0.8467 (0.8455-0.8477) |

*Notes: Models were trained using 2013-2020 births, threshold selection and validation were conducted using 2021 births, and final predictive performance was evaluated using 2022 births. Values are presented as point estimates with 200-bootstrap 95% confidence intervals.*
